# Gas of Particles Obeying the Monotone Statistics

**DOI:** 10.3390/e25071095

**Published:** 2023-07-21

**Authors:** Francesco Fidaleo

**Affiliations:** Dipartimento di Matematica, Università di Roma Tor Vergata, 00133 Roma, Italy; fidaleo@mat.uniroma2.it

**Keywords:** quantum statistical mechanics, thermodynamics, quantum probability

## Abstract

The present note is devoted to the detailed investigation of a concrete model satisfying the block-monotone statistics introduced in a previous paper (joint, with collaborators) of the author. The model under consideration indeed describes the free gas of massless particles in a one-dimensional environment. This investigation can have consequences in two fundamental respects. The first one concerns the applicability of the (block-)monotone statistics to concrete physical models, yet completely unknown. Since the formula for the degeneracy of the energy-levels of the one-particle Hamiltonian of a free particle is very involved, the second aspect might be related to the, highly nontrivial, investigation of the expected thermodynamics of the free gas of particles obeying the block-monotone statistics in arbitrary spatial dimensions. A final section contains a comparison between the various (block, strict, and weak) monotone schemes with the Boltzmann statistics, which describes the gas of classical particles. It is seen that the block-monotone statistics, which takes into account the degeneracy of the energy-levels, seems the unique one having realistic physical applications.

## 1. Introduction

Motivated by the fundamental investigation concerning the statistics of elementary particles in terms of suitable commutation relations between basic operators (e.g., [1]) like annihilators and creators, or equivalently field operators, the exploration of many other commutation relations has undergone an impetuous growth. Having in mind potential applications in physics, such a vast investigation has been started mainly due to the connection with *quantum probability*. Since the literature on such a topic is extremely extended, we mention only a short sample (e.g., [2,3,4]), which is very far from being complete. In this direction, we also cite the seminal paper [5], which proposes an interesting interplay between classical measure theory and noncommutative geometry with the aim of providing a fruitful treatment of interacting quantum fields. Finally, the reader is referred to the monograph [6] for an introduction to *Voiculescu free probability*, the latter being a relevant branch of quantum probability.

We recall that the description of Bose and Fermi particles in terms of commutation relations of operators acting on the corresponding Fock spaces encodes the statistics that the particles under consideration obey. Therefore, such a description in terms of commutation relations is also the main ingredient for the investigation of the thermodynamic properties of the infinitely extended gas of such primordial families of elementary particles. Just to mention some examples, with such a picture it is possible to provide a satisfactory description of the *Bose–Einstein condensate* responsible for the condensation in the ground state of integer spin particles like massive particles and photons (see [7] for the meaning of the condensation effect for such very special particles), and quasi-particles like phonons, and rotons entering in the description of superfluidity, see e.g., [8,9].

Concerning the half-integer spin particles like electrons, we mention the description of the *Pauli exclusion principle*, encoded in the corresponding Fermi commutation relations and relevant for the study of the statistical properties of metals and semiconductors. On the other hand, we point out that the thermodynamics of exotic particles obeying the statistics described by such exotic commutation relations (see [4] for such a family) has not yet been intensively investigated due to several reasons.

Recently, the attempt to systematically investigate the thermodynamic properties of such exotic particles has been carried out in the series of papers [10,11,12,13,14]. The first study was that concerning the *q-particles* or *quons*, q∈[−1,1]. They correspond to a mere interpolation between the Fermi case corresponding to q=−1 and the Bose one corresponding to q=1, passing from q=0, corresponding to the Boltzmann statistics (i.e., classical particles).

Notice that, in quantum mechanics, the indistinguishability of particles plays a crucial role. Since the statistics that the quons obey is completely unknown, the *q*-deformed Fock space cannot be used at all to compute the thermodynamic properties of such particles. This is the same for the Boltzmann case (see e.g., [15]). Since the statistics of classical particles is perfectly known, it is possible to correct the various statistical weights corresponding to the *n*-level occurring in the grand-partition function with the Gibbs correction 1/n!. Since n! is nothing but the number of permutations on *n* objects, such corrections take into account the indistinguishability of particles, see e.g., [16]. Nevertheless, the calculation of the grand-partition function for quons, which encodes all thermodynamic properties of an infinitely extended medium, has still been carried out in [11] without taking into account the possible statistics of such particles which are perhaps completely unknown.

Other relevant commutation relations are the *Boolean* and *monotone* ones. The Boolean scheme (e.g., [17]) describes the absorption/emission of a single photon by a medium, see [18]. Therefore, the Boolean Fock space is the simplest among the nontrivial ones, and thus the arising thermodynamic description presents no troubles.

By mainly looking at quantum probability, the monotone scheme was introduced in [19,20]. It is still unknown whether such a scheme can have potential applications to physics. Since the ergodic behaviour of any (classical or quantum) stochastic process is relevant for the investigation of its main properties (e.g., [21]), we refer the reader to [22] for a systematic investigation of the ergodic properties of such non conventional quantum stochastic processes.

The monotone scheme is based on a pre-assigned order on the basis of the (separable) one-particle Hilbert space H, and two possibilities were investigated in some detail. These are called the *strictly monotone* (simply called “monotone” in most of the related literature and in the present paper) and the *weakly monotone schemes*. On the other hand, many (but not all significant) meaningful physical models are described by a one-particle Hamiltonian *H* with compact resolvent, acting on a, typically separable, Hilbert space H. Therefore, there is a completely natural order on the necessarily discrete spectrum σ(H) of the Hamiltonian *H* and, in addition, the set of normalised eigenvectors of *H* provides an orthonormal basis of *H*. Unfortunately, the energy-levels can have a degeneracy, and thus no total pre-order is inherited on the basis of H made of eigenvectors of *H*. To overcome this problem in view of possible physical applications, in [13] a new scheme was introduced, called *block-monotone*, which takes into account the natural partial order on the basis of H made of eigenvectors of *H*, established by considering the possible degeneracy of the energy-levels.

Since it is almost impossible to provide an explicit calculation of the block-monotone grand-partition function for most reasonable models, in the previously mentioned paper [13], we analysed a simple model, perhaps without degeneracy (and thus for which monotone and block-monotone schemes coincide). For such a model, we see that the correction factor 1/n!, connected to indistinguishability, automatically appears in the computation of the statistical weights corresponding to the various *n*-levels. This suggests that the indistinguishability of the involved particles is automatically encoded in the monotone scheme. However, we point out that the role of the degeneracy was left out when managing that simple model.

The present paper was devoted to the study of the role of the degeneracy by analysing a simple real model for which it is nontrivial, and thus the monotone and the block-monotone differ from each other. We still checked that the Gibbs factor n! naturally appears for this more complicated situation, as well as the decimation with respect to the Boltzmann model. As in the previous model of [13], we explicitly estimated such a decimation in the case of a low density/high energy regime.

This paper ends with the comparison concerning the asymptotics in the high temperature regime (i.e., β≈0) of the statistical weights of Boltzmann, block-monotone, monotone and weak monotone particles. Concerning the leading terms, we see that all monotone schemes provide the same result, perhaps perfectly coinciding with the leading terms of the Boltzmann statistics. Therefore, one might conclude that all monotone schemes have reasonable physical applications. However, by looking at the successive terms in such an asymptotic expansion, one recognises that only the monotone scheme presents a natural decimation with respect to the Boltzmann case. Therefore, one can argue that only the block-monotone scheme might be suitable for potential physical applications.

## 2. Preliminaries

**One-particle Hamiltonian.** We deal with systems whose one-particle Hamiltonian *H* is a self-adjoint positive (i.e., σ(H)⊂[0,+∞)) operator with compact resolvent, acting on a separable Hilbert space H, which is nothing but the *one-particle space*. Therefore, the spectrum σ(H) is made of isolated points ε∈σ(H), with finite (possible non uniform) multiplicity g(ε), accumulating at +∞ if H is infinite dimensional.

Denoting by $P_{\varepsilon }$ the self-adjoint eigenprojector onto the eigenspace corresponding to the eigenvalue ε∈σ(H), we have for the resolution of the identity associated to *H*, $I\equiv I_{H}=\sum_{\varepsilon \in \sigma (H)}P_{\varepsilon }$, and

$$H=\sum_{\varepsilon \in \sigma (H)}\varepsilon P_{\varepsilon },\;\;\mathrm{with}\;\;g\left(\varepsilon \right)=\dim\left(\mathrm{Ran}(P_{\varepsilon })\right)<\infty \;.$$

Let $k_{B}\approx 1.3806488\times 10^{-23}\;JK^{-1}$ be the Boltzmann constant and $\beta :=\frac{1}{k_{B}T}$ the “inverse temperature”. We assume that $e^{-\beta H}$ is a trace class for each β>0 and define the *partition function* $\zeta :=\mathrm{Tr}(e^{-\beta H})$.

For many models describing the gas of massive and massless particles or quasi-particles confined in a finite volume in the *d*-dimensional space (for example, if d=1,2, the “volume” is indeed a length or a surface, respectively), we can compute the corresponding finite-volume partition and grand-partition functions.

For the purpose of the present note, we deal with the massless case. It is also customary to deal with the one-particle Hamiltonian obtained by imposing the periodic boundary conditions, which is given by

(1) $$H:=\frac{vh}{L}\left|k\right|\;.$$

Here, *L* is the one-dimensional volume (i.e., the length of the manifold $\frac{L}{2\pi }T$, T being the unit circle), equipped with periodic boundary conditions, *v* is the speed of the light in case of photons or the velocity of the sound in the medium in case of phonons. Therefore, $k:=z\frac{d}{dz}=\frac{1}{𝚤}\frac{d}{d\vartheta }$ ($z=e^{𝚤\vartheta }$, ϑ∈[0,2π)) is the momentum operator, whose spectrum is Z. We note that the degeneracy of the energy-levels of *H* is 2, but the non-degenerate ground state corresponding to k=0.

For the partition function ζ≡ζ(β), an easy computation yields

$$\zeta \left(\beta \right)=\sum_{k\in Z}e^{-\beta \frac{vh}{L}\left|k\right|}=2\sum_{k=0}^{+\infty }e^{-\beta \frac{vh}{L}k}-1=\frac{e^{\beta \frac{vh}{L}}+1}{e^{\beta \frac{vh}{L}}-1}=\mathrm{cotanh}\left(\frac{\beta vh}{2L}\right)\;.$$

Consequently, for the grand-canonical partition function $Z^{\left(o\right)}\equiv Z^{o}\left(\beta ,z\right)$ of infinitely many of such particles obeying the Boltzmann statistics, we get

(2) $$Z^{\left(o\right)}\left(\beta ,z\right)=e^{z\zeta (\beta )}=\sum_{n=0}^{+\infty }\frac{z^{n}}{n!}{\left(\frac{e^{\beta \frac{vh}{L}}+1}{e^{\beta \frac{vh}{L}}-1}\right)}^{n}\;.$$

**The grand-partition function.** Here, we define the *grand-partition function* in a relatively general framework relative to a gas comprising non-interacting particles obeying rather general statistics, and thus potentially suitable for physical applications. The knowledge of such grand-partition functions plays a crucial role in the so-called *equilibrium statistical mechanics*. The standard method for such an analysis is the so-called *second quantisation*, see, e.g., [1,16].

Indeed, for the one-particle Hilbert space H, we define the so-called *full Fock space*$F_{0}\left(H\right)\equiv F$, given by

$$F:=\overset{+\infty }{\underset{n=0}{⨁}}\underset{n\text{-}\mathrm{times}}{\underset{︸}{H⊗\cdots ⊗H}}\;,$$

with the convention that $\underset{0\text{-}\mathrm{times}}{\underset{︸}{H⊗\cdots ⊗H}}\;:=C\equiv C\Omega$, where Ω is the so-called *vacuum vector*.

The *number operator*, denoted by *N* with a slight abuse of notation, which “counts” the number of particles in the various levels of the Fock space, has a clear meaning (see e.g., [1]). It is easily defined on generators as

$$N\left(e_{j_{1}}⊗\cdots ⊗e_{j_{n}}\right):=n(e_{j_{1}}⊗\cdots ⊗e_{j_{n}})\;,$$

and extends by linearity to the whole own domain as a self-adjoint operator.

For a linear operator *A* with domain D⊂H, we define

$$\begin{array}{cc} d\Gamma_{o}{\left(A\right)\lceil }_{D⊗\cdots ⊗D}:= & A⊗I⊗\cdots ⊗I+I⊗A⊗\cdots ⊗I\\ +\cdots + & I⊗\cdots ⊗A⊗I+I⊗\cdots ⊗I⊗A\;, \end{array}$$

and extend it to its own domain in the whole Fock space by linearity. For *A* self-adjoint, the closure dΓ(A) of $d\Gamma_{o}\left(A\right)$ will be still self-adjoint, see, e.g., [1]. The simplest example is the 2nd quantised $d\Gamma (I_{H})$ of the identity $I_{H}$ of B(H), which is nothing but the number operator *N* defined above. Notice that dΓ(A) is unbounded in all situations (but A=0), even when *A* is bounded.

Let *P* be a self-adjoint projection acting on F. For a Hamiltonian *H* as above, the parameters β>0 (the inverse temperature) and μ∈R (the chemical potential) such that $Pe^{-\beta d\Gamma (H-\mu I)}P\equiv Pe^{-\beta (d\Gamma (H)-\mu N)}P$ is trace class, the *grand-partition function* is defined as

(3) $$Z_{P}\equiv Z_{H,P}\left(\beta ,\mu \right):=\mathrm{Tr}\left(Pe^{-\beta d\Gamma (H-\mu I)}P\right)\;.$$

It is customary to express any grand-partition function *Z* in terms of the *activity* (or *fugacity*) $z:=e^{\beta \mu }$. In all meaningful cases, *Z* admits the Mc Laurin expansion in the activity as

$$Z\left(\beta ,z\right)=\sum_{n=0}^{+\infty }a_{n}\left(\beta \right)z^{n}\;,$$

where $a_{0}\left(\beta \right)$ is always 1 and obviously, for n>1, $a_{n}\left(\beta \right)$ depends on the models under consideration.

Notice that (e.g., [16]) the average number of particles is given by $N=z\frac{\partial \;\;}{\partial z}\ln Z$. Therefore,

$$p_{n}\left(\beta ,z\right):=\frac{1}{Z(\beta ,z)}a_{n}\left(\beta \right)z^{n}$$

is nothing but the probability of finding *n* particles in thermodynamical equilibrium at fixed activity *z* and inverse temperature $\beta \equiv \frac{1}{k_{B}T}$. For this reason, the coefficients $a_{n}\left(\beta \right)$ are called *statistical weights*.

The most relevant cases describing the thermodynamics of Bose and Fermi gases are those when *P* is the self-adjoint projections onto the completely symmetric and antisymmetric subspaces (with respect to the natural action of the permutations on F), respectively.

With respect to $P=I_{F}$ corresponding to the grand-partition function of Boltzmann (or classical) particles, it was shown that this is not the case (e.g., [15]). However, we can still compute such a grand-partition function by correcting the statistical weight corresponding to *n*-particles with the factor 1/n!. In this situation, corresponding to the case of the free gas of classical particles, we have

$$Z_{\mathrm{Boltzmann}}=\sum_{n=0}^{+\infty }\frac{{\left(z\mathrm{Tr}e^{-\beta H}\right)}^{n}}{n!}=e^{z\zeta }$$

where, as before, $z=e^{\beta \mu }$ is the activity and $\zeta =\mathrm{Tr}e^{-\beta H}$ is the partition function relative to the Hamiltonian *H*.

Such a picture also takes into account the simplest Boolean situation, where $F_{\mathrm{boole}}=C\Omega ⨁H$, and thus $F_{\mathrm{boole}}=P_{\mathrm{boole}}F$, with

$$P_{\mathrm{boole}}=I_{C\Omega }⊕I_{H}⊕0_{H⊗H}⊕0_{H⊗H⊗H}⊕\cdots \;\;.$$

As explained in [13], it was shown that there is some evidence that such a picture could provide the grand-partition function of the (block-)monotone case. The present paper is devoted to confirming this evidence.

**Monotone Fock space.** For the reader’s convenience, we report some basic facts regarding monotone Fock spaces, see [2,19,20] for more details. To simplify, we considered the separable situation $H∼\ell^{2}\left(N\right)$ with the natural order on an orthonormal basis $\left\{e_{n}|n\in N\right\}$ (or any ordered basis of a separable Hilbert space). The monotone Fock space is then built as follows. The *n*-particle space is indeed spanned by the vectors $e_{\alpha }\equiv e_{j_{1}}⊗e_{j_{2}}⊗\cdots ⊗e_{j_{n}}$, whenever $\alpha =\{j_{1},j_{2},\cdots ,j_{n}\}\subset N$, $j_{1}<j_{2}<\cdots <j_{n}$ is any ordered string made of *n* elements. If we relax the last condition by merely assuming that $j_{1}\leq j_{2}\leq \cdots \leq j_{n}$, we will obtain the so-called *weakly monotone* Fock space, see, e.g., [2]. It is also customary to denote the monotone scheme as the *strict monotone* one to distinguish this from the weak monotone one.

Note that $F_{m}$ and $F_{\mathrm{wm}}$ are the range of the self-adjoint projections $P_{m}$ and $P_{\mathrm{wm}}$ acting on the full Fock space F:

$$F_{m}=P_{m}F\;\;\;\mathrm{and}\;\;\;F_{\mathrm{wm}}=P_{\mathrm{wm}}F\;,$$

where $P_{m}$ and $P_{\mathrm{wm}}$ project onto the subspaces spanned by the orthonormal elements described above.

**Block-Monotone Particles.** Here, we report the generalisation of the monotone scheme for the statistics of the particles previously introduced in [13] which, on the one hand, seems to be more suitable for potential physical applications and, on the other hand, is always different from the weak monotone and (strict) monotone schemes whenever the degeneracy of the energy-levels of the model under consideration is nontrivial.

For this purpose, we consider an index-set *I*, necessarily finite or countable, which is a finite or countable disjoint union of finite sets. This description comes from the natural order inherited on the basis

$$\left\{e_{\varepsilon }|\varepsilon \in \sigma \left(H\right)\right\}=\underset{\varepsilon \in \sigma (H)}{⨆}\left\{e_{\varepsilon ,j}|j=1,\cdots ,g\left(\varepsilon \right)\right\}$$

of H made of orthonormal eigenvectors of the Hamiltonian *H*.

Indeed, $I:={⨆}_{j=0}^{+\infty }I_{j}$, where $|I_{j}|<+\infty$, j=0,1,…. The set *I* is naturally partially ordered, because if $k_{j},l_{j}$ are in the same subset $I_{j}$, there is no pre-assigned order between them. Conversely, if $k_{1}\in I_{j_{1}}$ and $k_{2}\in I_{j_{2}}$, then $k_{1}≺k_{2}\Leftrightarrow j_{1}<j_{2}$. The block-monotone *n*-particle subspace is then generated by all sequences of the elementary (orthonormal) tensors $e_{k_{1}}⊗\cdots e_{k_{n}}$ with the condition $k_{1}<k_{2}<\cdots <k_{n}$ relative to the partial order defined above.

This picture is suggested by potential physical applications. In fact, a positive Hamiltonian *H* with compact resolvent acting on a separable Hilbert space H induces a natural order, as shown above, on the natural basis of H made of the eigenvectors of *H*. Such a scheme is defined as *block-monotone.* The corresponding block-monotone Fock space $F_{\mathrm{bm}}$ is easily constructed as follows.

Indeed, let $\left\{e_{j}|j\in I\right\}$ be an orthonormal basis of H equipped with the previously described partial order. In our framework, such a partial order is induced by a positive Hamiltonian with compact resolvent as previously described. We also note that $F_{\mathrm{bm}}$ is a subspace of $F_{m}$, which is proper if and only if the degeneracy of the energy-levels is nontrivial. In other words, the block-monotone scheme coincides with the monotone one if and only if the energy-levels are nondegenerate (or, equivalently, the $I_{j}$ are singletons).

Obviously, both subspaces $F_{\mathrm{bm}}$ and $F_{m}$ are subspaces of the full Fock one $F\equiv F_{0}$, which are always proper whenever dim(H)>1.

The corresponding annihilator and creator operators acting on the block-monotone Fock space can also be easily constructed. Since we do not use such operators here, we leave the details to the interested reader.

## 3. The Grand-Partition Function of the Free Gas of Monotone Particles

In order to compute the block-monotone grand-partition function $Z^{\left(\mathrm{bm}\right)}\left(z,\beta \right)$, we reason as in [13]. This is certainly more complicated than the degeneracy-free models.

Indeed, taking into account that the degeneracy of the levels $g(\varepsilon_{k})$, $\varepsilon_{k}=\frac{vh}{L}\left|k\right|$, and k∈Z, is obviously

$$g\left(\varepsilon_{k}\right)=\left\{\begin{array}{cc} 1 & \mathrm{if}\;\;k=0\;,\\ 2 & \mathrm{otherwise}\;, \end{array}\right.$$

we obtain

$$\begin{array}{cc} \mathrm{Tr} & (P_{\mathrm{bm}}e^{-\beta d\Gamma (H)}P_{\mathrm{bm}}\lceil \underset{n\text{-}\mathrm{times}}{\underset{︸}{H⊗\cdots ⊗H}})=2^{n}\sum_{k_{1}=1}^{+\infty }e^{-\beta \frac{vh}{L}k_{1}}\sum_{k_{2}=k_{1}+1}^{+\infty }e^{-\beta \frac{vh}{L}k_{2}}\\ \; & \cdots \cdots \sum_{k_{n}=k_{n-1}+1}^{+\infty }e^{-\beta \frac{vh}{L}k_{n}}+2^{n-1}\sum_{k_{1}=1}^{+\infty }e^{-\beta \frac{vh}{L}k_{1}}\sum_{k_{2}=k_{1}+1}^{+\infty }e^{-\beta \frac{vh}{L}k_{2}}\\ \; & \cdots \cdots \sum_{k_{n-1}=k_{n-2}+1}^{+\infty }e^{-\beta \frac{vh}{L}k_{n-1}}=2^{n-1}\left(e^{\beta \frac{vh}{L}n}+1\right)\prod_{k=1}^{n}\frac{1}{e^{\beta \frac{vh}{L}k}-1}\;. \end{array}$$

Consequently, for z≥0 and β>0, we have

(4) $$Z^{\left(\mathrm{bm}\right)}\left(z,\beta \right)=1+\sum_{n=1}^{+\infty }{\left(2z\right)}^{n}\frac{e^{\beta \frac{vh}{L}n}+1}{2}\prod_{k=1}^{n}\frac{1}{e^{\beta \frac{vh}{L}k}-1}.$$

**Proposition 1.**  *For the grand-partition function* $Z^{\left(\mathrm{bm}\right)}\left(z,\beta \right)$ *in (4), we have*

(5) $$0\leq Z^{\left(\mathrm{bm}\right)}\left(z,\beta \right)\leq Z^{\left(o\right)}\left(2z,\beta \right)\;,$$

*where $Z^{\left(o\right)}$ is the Boltzmann grand-partition function given in (2), and thus $Z^{\left(\mathrm{bm}\right)}$ converges for all z≥0 and β>0.*

**Proof.**  We first note (cf. [13]) that, for β>0,

$$\prod_{k=1}^{n}\frac{1}{e^{\beta \frac{vh}{L}k}-1}<\frac{1}{n!(e^{\beta \frac{vh}{L}}-1{)}^{n}}\;.$$

On the other hand, a coarse approximation yields

$$\frac{e^{\beta \frac{vh}{L}n}+1}{2}<{\left(e^{\beta \frac{vh}{L}}+1\right)}^{n}\;.$$

Collecting these together, we get (5). □

In the next section, we discuss a finer inequality than that in (5).

By coming back to the applicability of various monotone statistics to realistic physical models, the previous analysis yields that the block-monotone prescription could certainly be suitable. We also note that, for the statistical weights we easily have

$$a_{n}^{\left(\mathrm{wm}\right)}\geq a_{n}^{\left(m\right)}\geq a_{n}^{\left(\mathrm{bm}\right)}\;,$$

independently on the degeneracy of the involved energy-levels.

## 4. Comparison with the Boltzmann Particles

The key-point with which to compare the thermodynamic properties of monotone and Boltzmann particles is to provide an inequality finer than that in (5). In order to do that, we follow the lines in [13], Section 3. At this stage, we provide only a qualitative analysis (perhaps confirmed with the aid of software symbolic calculi), leaving the insights to the interested scientist.

More precisely, the first part of the analysis follows the same lines of the analogous part in [13], Proposition 2, the latter corresponding to (9). The final part of the present section concerning (11) (to be compared with the analogous Equation (17) in [13]) provides only a qualitative estimate of the decimation phenomenon arising in monotone statistics.

We start with the analogy of [13], Proposition 3.1 and, with

$$Z^{\left(\mathrm{bm}\right)}=\sum_{n=0}^{+\infty }a_{n}^{\left(\mathrm{bm}\right)}\;,\;Z^{\left(o\right)}=\sum_{n=0}^{+\infty }a_{n}^{\left(o\right)}\;,$$

$x:=e^{\beta \frac{vh}{L}}$, we argue that

(6) $$a_{n}^{\left(o\right)}-a_{n}^{\left(\mathrm{bm}\right)}=\frac{1}{n!}{\left(\frac{x-1}{x+1}\right)}^{n}\left(\Delta_{n}\left(1\right)+\Delta_{n}^{\prime }\left(1\right)\left(x-1\right)+\frac{\Delta_{n}^{\prime \prime }\left(\xi_{n}\right)}{2}{\left(x-1\right)}^{2}\right)\;.$$

In the situation under consideration,

(7) $$\Delta_{n}\left(x\right):=1-\frac{n!2^{n-1}\left(1+x^{n}\right)}{\left(1+x\right)\left(1+x+x^{2}\right)\cdots \left(\sum_{k=0}^{n-1}x^{k}\right)}\;,\;n=1,2,\ldots \;,$$

and $\xi_{n}$ is some number (depending obviously on *n*) in (1,x). As in [13], $\Delta_{n}\left(1\right)=0$ and we have the evidence that $\Delta_{n}^{\prime \prime }\left(\xi_{n}\right)<0$ whenever x>1. Quite surprisingly, also in this situation, $\Delta_{n}^{\prime }\left(1\right)=\frac{n(n-1)}{4}$. With $y:=\frac{x+1}{x+1}$, we reason as in the previously mentioned paper, obtaining

(8) $$\begin{array}{cc} Z^{\left(o\right)}-Z^{\left(\mathrm{bm}\right)}\leq & \frac{\left(x-1\right)}{4}\sum_{n=0}^{+\infty }n\left(n-1\right)\frac{y^{n}}{n!}=\frac{\left(x-1\right)}{4}y^{2}\frac{d^{2}Z_{0}}{dy^{2}}\\ = & \frac{z^{2}{\left(x+1\right)}^{2}}{4(x-1)}e^{y}=\frac{z^{2}{\left(x+1\right)}^{2}}{4(x-1)}Z_{0}\;. \end{array}$$

Collecting altogether, with

$$f\left(\beta ,z\right):=\frac{1}{e^{\beta \frac{vh}{L}}-1}{\left(\frac{z\left(e^{\beta \frac{vh}{L}}+1\right)}{2}\right)}^{2}\;,$$

we get the more refined inequalities

(9) $$\left(1-f\left(\beta ,z\right)\right)Z^{\left(o\right)}\left(z,\beta \right)\leq Z^{\left(\mathrm{bm}\right)}\left(\beta ,z\right)\leq Z^{\left(o\right)}\left(z,\beta \right)\;.$$

We note that (9) allows us to show

(10) $$\lim_{\overset{\left(\beta ,z)\to (0,0\right)}{(\beta ,z)\in R}}\frac{Z^{\left(\mathrm{bm}\right)}\left(\beta ,z\right)}{Z^{\left(o\right)}\left(\beta ,z\right)}=1\;,$$

where *R* is a suitable “sectorial” region in the (z,β)-1st quadrant having (0,0) as a cluster point, and thus describing the *low density*/*high temperature regime*.

In such a situation of low density/high temperature regime, (9) yields, at least in a rough but realistic approximation,

(11) $$Z^{\left(\mathrm{bm}\right)}\left(\beta ,z\right)\approx \left(1-f\left(\beta ,z\right)\right)Z^{\left(o\right)}\left(\beta ,z\right)\;,$$

where the minus sign, holding for (β,z)∈R, explains the decimation with respect to the classical case arising from the monotone statistics. We note that such a decimation is analogous to the *Pauli exclusion principle* for Fermi particles.

As explained in [13], such a *monotone exclusion principle* is manifestly relevant in the high-density regime corresponding to β↑+∞. Conversely, according to one of the early principles of thermodynamics asserting that all free gases of particles must have the same behaviour in the low density/high temperature regime independently of the statistics they obey (e.g., (10)), the monotone exclusion principle tends to be negligible in the low-density regime as expected. In the low-density regime, 1−f(β,z) provides a qualitative estimate of the correction corresponding to the monotone case with respect to the analogous ones relative to the Boltzmann one.

We note that, by (11), it is possible to determine the qualitative correction (with respect to the Boltzmann case) to all natural thermodynamic potentials. Indeed, for the average number of particles *N* with (β,z)→(0,0) in the region *R*,

$$\begin{array}{cc} N^{\left(\mathrm{bm}\right)}\left(\beta ,z\right)= & z\frac{\partial \ln Z^{\left(\mathrm{bm}\right)}\left(\beta ,z\right)}{\partial z}\approx z\frac{\partial \ln(1-f(\beta ,z))}{\partial z}+N^{\left(o\right)}\left(\beta ,z\right)\\ = & N^{\left(o\right)}\left(\beta ,z\right)-\frac{2{\left(e^{\beta \frac{vh}{L}}+1\right)}^{2}z^{2}}{4\left(e^{\beta \frac{vh}{L}}-1\right)-{\left(e^{\beta \frac{vh}{L}}+1\right)}^{2}z^{2}}\;. \end{array}$$

## 5. Comparison between Various Monotone Statistics

Concerning the various monotone statistics, it seems a delicate question to decide which of these is (more) suitable for concrete physical applications.

Following one of the main principles of thermodynamics, which asserts that all involved particles must be considered indistinguishable, this issue can be tested in the high temperature regime, that is, when β≈0. Similarly to the previous paper [13], it is then natural to compare the asymptotics of the various statistical weights,

(12) $$\begin{array}{cc} \{a_{n}^{\left(\#\right)}|n\in N,\;\;\#\;\;\; & \mathrm{standing}\;\mathrm{for}\;\mathrm{Boltzmann},\;\mathrm{monotone},\\ \; & \mathrm{block}\text{-}\mathrm{monotone},\;\mathrm{weak}\;\mathrm{monotone}\;\}. \end{array}$$

To simplify, we consider the Hamiltonian such that σ(H)={n+1∣n∈N}, with uniform degeneracy $g(\varepsilon_{n})=2$ for all *n*.

First of all, concerning the leading term of the expansion of the $a_{n}^{\left(\#\right)}$, it is not hard to show that

$$a_{n}^{\left(\#\right)}=\frac{2^{n}}{n!\beta^{n}}\left(1+o(1)\right)\;\;\mathrm{for}\;\;\beta \approx 0\;,$$

for all models in (12). Therefore, all such models obeying the various monotone statistics seem to be suitable for potential physical applications. We can then deduce that, in order to appreciate the difference between the various models, we should consider the high energy expansion of all statistical weights $a_{n}^{\left(\#\right)}$. We point out that such a process of expansion seems to be a quite natural one in investigating and solving long-standing problems of quantum physics, see [23]. The reader is also referred to [12] for a situation more close to the topics of the present paper.

For the convenience of the reader, we report the computations relative to the statistical weights $a_{2}^{\left(\#\right)}$. The results of such computations are listed below.

(13) $$\begin{array}{cc} a_{2}^{\left(o\right)}\left(\beta \right)= & \frac{\zeta {\left(\beta \right)}^{2}}{2}=\frac{2}{{\left(e^{\beta }-1\right)}^{2}}=\frac{2}{\beta^{2}}\left(1-\beta +o(\beta )\right)\;,\\ a_{2}^{\left(\mathrm{bm}\right)}\left(\beta \right)= & 4\sum_{k_{1}=1}^{+\infty }e^{-\beta k_{1}}\sum_{k_{2}=k_{1}+1}^{+\infty }e^{-\beta k_{2}}=\frac{4}{\left(e^{2\beta }-1\right)\left(e^{\beta }-1\right)}\\ \; & \;\;=\frac{2}{\beta^{2}}\left(1-\frac{3}{2}\beta +o\left(\beta \right)\right)\;,\\ a_{2}^{\left(m\right)}\left(\beta \right)= & a_{2}^{\left(\mathrm{bm}\right)}\left(\beta \right)+\frac{1}{e^{2\beta }-1}=\frac{2}{\beta^{2}}\left(1-\frac{1}{2}\beta +o\left(\beta \right)\right)\\ a_{2}^{\left(\mathrm{wm}\right)}\left(\beta \right)= & 4\sum_{k_{1}=1}^{+\infty }e^{-\beta k_{1}}\sum_{k_{2}=k_{1}}^{+\infty }e^{-\beta k_{2}}=\frac{4e^{3\beta }}{\left(e^{2\beta }-1\right)\left(e^{\beta }-1\right)}\\ \; & \;\;=\frac{2}{\beta^{2}}\left(1+\frac{3}{2}\beta +o\left(\beta \right)\right)\;. \end{array}$$

Summarising,

$$a_{2}^{\left(\mathrm{bm}\right)}\left(\beta \right)\overset{<}{∼}a_{2}^{\left(o\right)}\left(\beta \right)\overset{<}{∼}a_{2}^{\left(m\right)}\left(\beta \right)\overset{<}{∼}a_{2}^{\left(\mathrm{wm}\right)}\left(\beta \right)\;\;\mathrm{for}\;\;\beta \approx 0\;.$$

We note that the unique model exhibiting a decimation at the level of the 2-particle subspace, with respect to the Boltzmann one, is indeed the block-monotone one as expected. Therefore, the latter seems to be uniquely adapted for reasonable physical applications.

## 6. Conclusions

The (anti)commutation relations were the milestone for managing models arising from elementary particle physics, and thus contain particles that obey Fermi and Bose statistics. Such commutation relations indeed encode the statistics of the elementary particles split into fermions and bosons corresponding to q=±1. Models that interpolate the commutation relations for fermions and bosons (mainly for −1<q<1) were also intensively investigated.

Recently, the study of models arising from many exotic commutation relations (cf. [4]) has undergone an impetuous growth, and hundreds of papers have been devoted to this argument. All these papers fall under the topic of so-called quantum probability, even if the possible physical applications were cited among the main motivations for such an investigation. To the best knowledge of the author, no physical applications have been described for such exotic models, except for the so-called Boolean one, which directly arises from the description of the phenomenon of the interaction between radiation and matter (cf. [18]).

Concerning the *q*-particles, the first concrete obstruction to their applicability to physics was pointed out in [15]. Indeed, it was shown that the grand-partition function corresponding to the Boltzmann case q=0, describing classical particles and computed by using the full Fock space, differs to that expected. This is certainly due to the indistinguishability of particles not encoded in the construction of the full Fock space.

Second, the grand-partition function for all −1<q<1, computed by using the *q*-Fock space (which is a deformation of the full Fock space corresponding to q=0), is independent of *q*. This paradox was solved in [11].

Concerning such *q*-particles, they appear as a mere interpolation between fermions (q=−1) and bosons (q=1), where q=0 corresponds to the classical particles. Therefore, excluding the cases ±1 and 0, the physical application of models describing quons seems, at the moment at least, to be wishful thinking.

The monotone statistics was invented and investigated in the quantum probability setting, after mentioning potential physical applications. Any model or, more precisely, the corresponding Fock space encoding the statistics that the model under consideration obeys, must satisfy some basic prescriptions. The most important one is that it must encode the indistinguishability of (necessarily quantum) particles. To the best knowledge of the author, no concrete application of the monotone model to physics is known. In addition, there has been no discussion about the suitability of such a model for concrete physical applications.

The first discussion on this relevant question started in [13], in which it was shown that the indistinguishability of particles appears to be automatically encoded, at least after the investigation of a simple model. The above mentioned paper also contains some realistic physical applications of the monotone statistics (Section 6), to which the interested reader is referred.

In the present note, we have continued the investigation of the monotone statistics for more complicated models. We explained which of the various monotone schemes may be suitable for realistic physical applications. The block-monotone scheme was previously introduced in [13] for this purpose. As mentioned above, some of the potential physical applications were also outlined.

Since the main object in the thermodynamics of the equilibrium, the grand-partition function, is not explicitly computable for all realistic models obeying monotone statistics, the question described above does not have an easy solution. On the other hand, the block-monotone model, which seems to be the right candidate for such physical applications, presents the additional difficulty (not considered in the previous paper [13]) of the nontrivial degeneracy—possibly non homogeneous—of the energy-levels of the one-particle Hamiltonian of the system under consideration.

In the present paper, after studying a realistic model with non homogeneous degeneracy, among other things, we have shown that the block-monotone statistics indeed seems to be the right model for this purpose. We showed that, at level of the statistical weights, only this scheme presents the expected decimation effect with respect to the classical model, see Section 5.

After these preliminary but fundamental considerations, the next step will be the systematic investigation of the gas of monotone massive and massless particles, necessarily in the block-monotone version and in arbitrary spatial dimensions. However, we point out that it will certainly be a very difficult task in the massive case, due to the explicit computability of the various statistical weights (i.e., some special functions, which cannot be analytically expressed, would enter into such computations). Another difficulty will be the very complicated behaviour of the degeneracy for dimensions ≥2 in both massive and massless cases, see, e.g., [13], Section 6.

## Data Availability

Not applicable.
